# Direct measurement of vertical forces shows correlation between mechanical activity and proteolytic ability of invadopodia

**DOI:** 10.1126/sciadv.aax6912

**Published:** 2020-03-11

**Authors:** E. Dalaka, N. M. Kronenberg, P. Liehm, J. E. Segall, M. B. Prystowsky, M. C. Gather

**Affiliations:** 1 SUPA, School of Physics and Astronomy, University of St Andrews, St Andrews, UK.; 2 Albert Einstein College of Medicine, Bronx, NY 10461, USA.

## Abstract

Mechanobiology plays a prominent role in cancer invasion and metastasis. The ability of a cancer to degrade extracellular matrix (ECM) is likely connected to its invasiveness. Many cancer cells form invadopodia—micrometer-sized cellular protrusions that promote invasion through matrix degradation (proteolysis). Although it has been hypothesized that invadopodia exert mechanical force that is implicated in cancer invasion, direct measurements remain elusive. Here, we use a recently developed interferometric force imaging technique that provides piconewton resolution to quantify invadopodial forces in cells of head and neck squamous carcinoma and to monitor their temporal dynamics. We compare the force exerted by individual protrusions to their ability to degrade ECM and investigate the mechanical effects of inhibiting invadopodia through overexpression of microRNA-375. By connecting the biophysical and biochemical characteristics of invadopodia, our study provides a new perspective on cancer invasion that, in the future, may help to identify biomechanical targets for cancer therapy.

## Teaser

A microscopy technique based on interference of light measures the piconewton forces that cancer cells exert during invasion.

## INTRODUCTION

Cellular forces are crucial for the coordination of many cellular processes, including cancer progression, invasion, and metastasis (*1*–*3*). The compliance of cells and of the surrounding extracellular matrix (ECM) and the mechanical action of cells on their surroundings all contribute to the malignant phenotype, e.g., via changes of cellular conformation and regulation of invasive and metastatic behavior. Of particular importance for the invasiveness of a cancer is its ability to degrade the ECM (*4*). Many types of cancer cells form invadopodia, specialized actin-rich protrusions to the cell membrane that are capable of ECM degradation (*5*, *6*). It has been hypothesized but not demonstrated that invadopodia exert mechanical force and that this force is implicated in cancer invasion (*7*).

The formation and activity of invadopodia include precursor assembly, anchoring, and maturation, the latter of which is accompanied by ECM degradation (*8*, *9*). Several proteins are involved in invadopodia formation, including actin, cortactin, and the tyrosine kinase adaptor proteins Tks5 and Tks4. The main invadopodium core consists of actin filaments; cortactin binds directly to these filaments and promotes key invadopodium functions, such as initiation, formation, and proteolytic behavior (*10*, *11*). Tks5 is essential for invadopodium formation and stability but not for matrix degradation, whereas Tks4 regulates the formation of invadopodial protrusions (*12*). ECM degradation occurs, at least in part, through the release of different matrix metalloproteases (MMPs), including membrane type 1 (MT1)–MMP, MMP-2, and MMP-9 (*13*).

Mechanical stimulation of cells has been correlated with higher numbers and enhanced proteolytic activity of invadopodia (*14*). There is also evidence that invadopodia protrude into their substrate in a dynamic, oscillatory way by actin polymerization (*15*) and possibly by actomyosin contractility (*16*) and that they perform local mechanosensing through the biochemical activity of integrins (*17*). Whole-cell contractility, measured via traction force microscopy (TFM), has been correlated with invadopodial activity to assess the metastatic ability of cells (*18*, *19*). However, direct observation and measurement of forces exerted by individual invadopodia remain elusive, with evidence for the existence of a mechanical force limited to the observation of nuclear indentations caused by invadopodia that are located between the ventral cell membrane and the nucleus (*20*).

A number of nonmalignant cells, including macrophages and endothelial cells, form processes known as podosomes, which are structurally and functionally related to invadopodia. Using either a modified form of atomic force microscopy (AFM) or elastic resonator interference stress microscopy (ERISM; see below), it was shown that podosomes exert dynamic vertical forces against the cell substrate that lead to an oscillatory protrusive behavior (*21*–*25*). Compared to podosomes, invadopodia are smaller in size, occur in lower numbers per cell (typically <20), and show a lower rate of turnover (*26*). Invadopodia generally form irregular patterns underneath the nucleus, while podosomes tend to arrange in more ordered, ring-shaped structures. This difference suggests that invadopodia can achieve mechanical support for protrusion via pushing against the cellular nucleus, while podosomes are directly linked to the actin cloud. The specific localization of invadopodia within the cell may also be functionally linked to the requirement to translocate the nucleus in a dense ECM (*27*). Yet, the question arises whether the degrading behavior of invadopodia is connected to a particular mechanical phenotype and whether ECM degradation is mechanically assisted.

Conventional stress microscopy techniques, such as TFM, generally reach at best a vertical stress sensitivity and spatial resolution of 50 Pa and 2 μm, respectively (*28*–*31*), and thus are unlikely to allow observation of the force exerted by individual invadopodia. Our recently introduced ERISM method monitors cell forces with high spatial resolution and unprecedented sensitivity to vertical forces, resolving vertical deformations of 2 nm, stresses of 1 Pa, and—for localized indentations—forces of ≈1 pN (*23*, *32*). This is achieved by growing cells on an optical microcavity substrate that consists of a layer of an ultrasoft elastomer that is situated between two semitransparent, mechanically flexible gold mirrors, the upper of which is coated with ECM proteins. Mechanical forces exerted by cells cause local deformations of the microcavity and, thus, local shifts to its resonance wavelengths. The resulting interference patterns are analyzed by optical and subsequent mechanical modeling to compute a high-resolution stress map. Unlike many other stress microscopy techniques, ERISM does not require a zero-stress reference image, allowing the experimenter to keep cells on the substrate for time-lapse imaging and immunostaining.

Here, we report the direct observation and quantification of invadopodial forces. By combining ERISM with a degradation assay, we compared the forces exerted by invadopodia to their proteolytic activity, i.e., ability to degrade ECM. Immunostaining was used to ascertain that the investigated structures are invadopodia. In addition, by studying the temporal evolution of invadopodial forces, we found an oscillatory protruding behavior with two characteristic time scales. Interfering with invadopodial protrusions by overexpression of the microRNA-375 (miR-375) resulted in reduced invadopodia force and impaired dynamics, indicating possible routes to target invadopodia-mediated cancer invasion.

## RESULTS

### Measurement of mechanical forces exerted by invadopodia

**Fig. 1:**
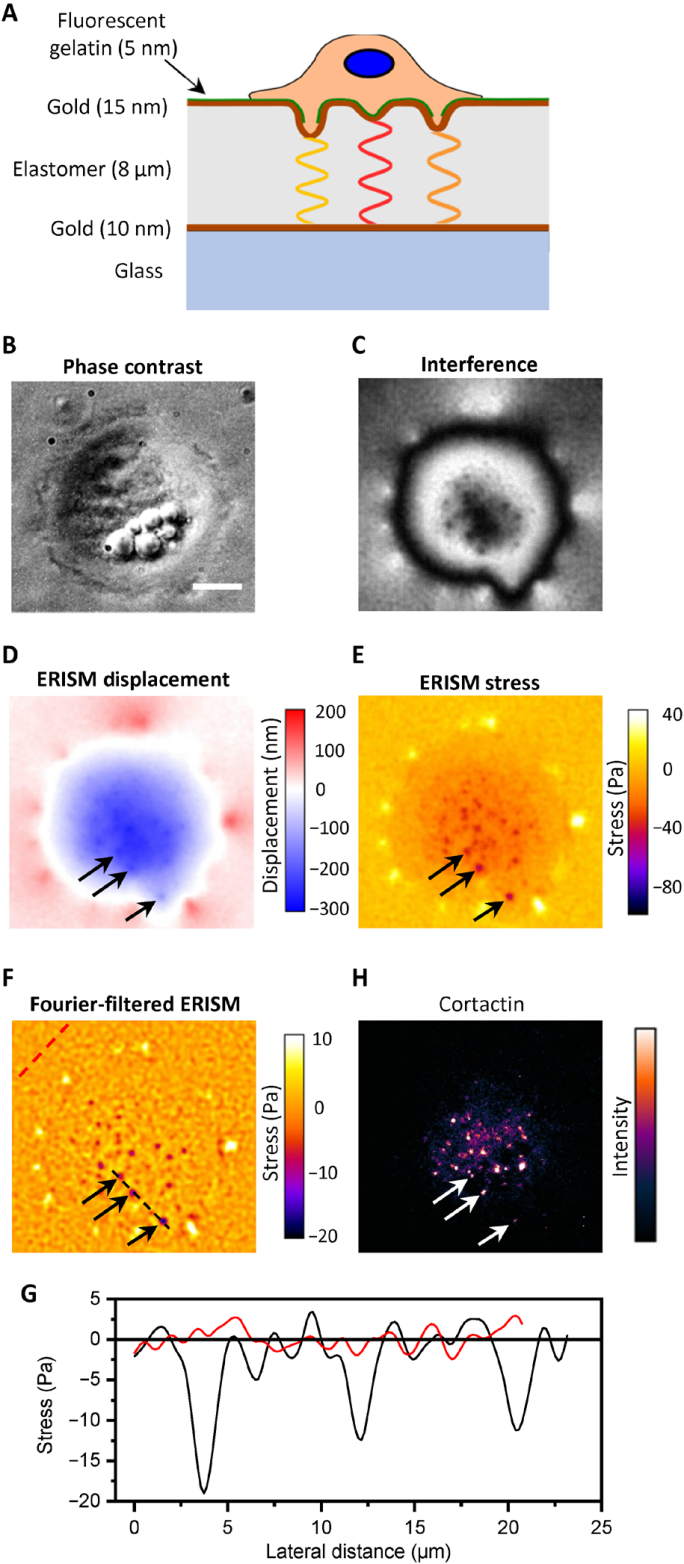
ERISM for measuring the mechanical activity of invadopodia formed by UM-SCC-1 cancer cells. (**A**) Schematic sketch of the ERISM measurement, illustrating how invadopodia forces against an elastic microcavity, formed by two gold films sandwiching a soft elastomer, lead to local changes in the thickness and resonance wavelengths of the microcavity. The microcavity substrate is coated with fluorescent gelatin (green line) to distinguish mature (degrading) from immature (nondegrading) invadopodia. (**B**) Phase-contrast image of UM-SCC-1 cell attached to the microcavity surface. (**C**) Coregistered monochromatic reflectance image of the microcavity, for illumination with 676-nm light. (**D**) ERISM thickness map obtained from a series of reflectance images, showing the substrate deformation induced by the same cell. Black arrows indicate three local indentation features. (**E**) ERISM stress map and (**F**) ERISM stress map after application of Fourier band-pass filter. (**G**) Lateral stress profile along the black and red dashed lines in (F). (**H**) Epifluorescence image of the same cell after immunostaining for cortactin. Scale bar, 10 μm.

Our studies used the UM-SCC-1 cell line, which was derived from head and neck squamous carcinoma (HNSCC) (*33*, *34*). HNSCC is an aggressive cancer that develops at the epithelial cell layer of the upper aerodigestive tract and is characterized by poor patient prognosis and low survival rates (*35*, *36*). Figure 1A shows a schematic of a UM-SCC-1 cell plated on an ERISM microcavity coated with collagen or fluorescent gelatin, illustrating how invadopodia may generate protrusions, exert force, and, thus, indent the ERISM microcavity, and how—depending on the amount of indentation—light of different wavelengths fulfills the resonance condition within the microcavity. Mature invadopodia are expected to degrade the underlying collagen or gelatin matrix, while immature invadopodia may still exert mechanical force even in the absence of matrix degradation. The ERISM substrates used in this work had an effective stiffness of 3 to 4 kPa (fig. S1), similar to the typical stiffness of tissue surrounding HNSCCs (*37*, *38*).

UM-SCC-1 cells attached well on collagen-coated ERISM substrates (Fig. 1B). Under illumination with monochromatic light, ERISM substrates showed a characteristic interference pattern, with large-scale features that map out the outer contour of the cell and typically 5 to 10 smaller-scale features underneath the cell (Fig. 1C). To quantify the deformation and stress associated with these features, we imaged the microcavity under illumination with monochromatic light of a range of different wavelengths and extracted the local deformation of the microcavity from these images via optical modeling (Fig. 1D). This revealed a broad indentation across the cell body and upward pulling along the cell periphery, a feature that has previously been associated with the presence of contractile forces, which are transmitted via focal adhesions (*23*). Superimposed on the broad indentation are local protrusions with a depth of approximately 6 nm. The deformation map was translated into a stress map via finite element modeling (Fig. 1E), and spatial Fourier filtering was applied to eliminate the unspecific stress from focal adhesions and overall cell contractility (Fig. 1F). This showed that the small protrusions are associated with local points of downward pushing. Integrating the stress over the area of each protrusion yielded a force of 2 to 10 pN per protrusion (see below for statistical analysis). Comparing the lateral stress profile along protrusions to background fluctuations in stress away from the cell shows that the recorded stress is well above the noise floor of the measurement (Fig. 1G).

To test whether the protrusive structures imaged with ERISM were due to invadopodial activity, we stained for actin and the invadopodia marker cortactin (Fig. 1H and fig. S2). Most protrusions correlated with actin and cortactin staining, corroborating that these result from invadopodia. We have previously found that the characteristic cortactin dots observed here colocalize fully with the invadopodia marker Tks5 (*34*), which we take as additional evidence that the observed protrusions are caused by invadopodia. Colocalization of cortactin, Tks5, and protrusive events was also confirmed for one example of cells on an ERISM substrate (fig. S2). Some cortactin puncta did not show a corresponding indentation. These nonindenting cortactin dots may be associated with early cortactin recruitment in developing invadopodial structures, i.e., with precursor assembly (*39*).

### Correlation of invadopodial force with ECM degradation

**Fig. 2:**
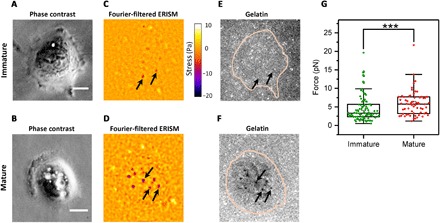
Correlation of invadopodial force with matrix degradation. (**A** and **B**) Phase-contrast images of two representative UM-SCC-1 cells with immature and mature invadopodia, respectively. (**C** and **D**) Fourier-filtered ERISM stress map of same cells, with arrows indicating representative invadopodia in each cell. (**E** and **F**) Epifluorescence image of fluorescent gelatin film on the surface of the ERISM substrate. Pink lines indicate the outline of cells. The maturity of invadopodia was classified by analysis of local gelatin fluorescence. (**G**) Comparison between force exerted by mature (*n* = 66) and immature (*n* = 92) invadopodia. Each data point represents the mean force exerted by an invadopodium over a 1-hour time frame. Plots indicate data (diamonds), median (center), mean (open square), Q1/Q3 (box), and ±1.5 SD (whiskers). As data in groups were not normally distributed, groups were compared using the Mann-Whitney *U* test (***P < 0.001). Scale bars, 10 μm.

Not all invadopodia formed by a cancer cell become mature, i.e., are able to degrade the ECM. To explore the forces exerted by mature and immature invadopodia separately, we determined invadopodial maturity with an accepted degradation assay that is based on a fluorescent gelatin layer that only mature invadopodia are able to degrade (*34*). In the literature, immunostaining for MT1-MMP is described as an alternative way to distinguish mature and immature invadopodia. Both assays provide similar results (*9*), and we opted for the fluorescent gelatin assay as this does not require fixation of cells and, thus, offers better compatibility with the live force imaging capability of ERISM. The ERISM substrate was coated with a thin film of fluorescent gelatin (thickness measured with AFM, 5.5 ± 1.2 nm), which did not cause a change in microcavity stiffness (fig. S1). Cells adhered well to the ERISM substrate (Fig. 2, A and B) and again showed the localized protrusions we previously associated with invadopodia activity (Fig. 2, C and D). Gelatin fluorescence was strongly decreased in some, but not all, of these tightly localized spots (Fig. 2, E and F), consistent with some invadopodia entering their maturation stage and degrading the matrix. Protrusions on ERISM maps that were colocalized with areas of degraded matrix were labeled as mature invadopodia, while protrusions without associated matrix degradation were classified as immature. We analyzed 14 cells and a total of 158 invadopodia, 66 of which were classified as mature (data from three independent experiments). Mature invadopodia exerted significantly larger forces than immature ones (median force ±SEM, *F*_mat_ = 5.8 ± 0.4 pN, *F*_imat_ = 3.4 ± 0.4 pN, *P* = 0.0008, Mann-Whitney *U* test; Fig. 2G). These data indicate that invadopodial force generation may play a role in ECM degradation, possibly supporting the enzymatic activity of MMPs (fig. S3). Comparing the forces exerted by mature and immature invadopodia at different time points, we found that at the single-cell level, only mature invadopodia become stronger as time evolves (fig. S4).

### Invadopodia exert highly dynamic and oscillatory forces

**Fig. 3:**
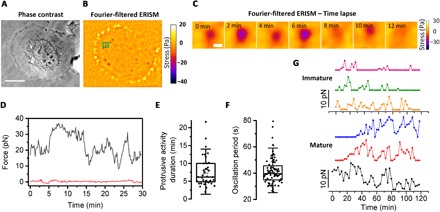
Dynamics of mechanical activity of invadopodia. (**A**) Phase-contrast image and (**B**) Fourier-filtered ERISM stress map of a typical UM-SCC-1 cell. (**C**) Time lapse of the invadopodium indicated by the green rectangle in (B), imaged every 2 min. (**D**) Temporal evolution of force exerted by a single invadopodium (gray line) and background force in a region without invadopodia (red line). Force measurements were taken every 10 s; recording started 2 hours after cell seeding. (**E**) Overall duration of protrusive activity and (**F**) mean duration of fast oscillation cycles in force for multiple invadopodia (3 individual experiments, 5 cells, and 93 invadopodia, of which 45 were classified as mature, 8 as immature, and the remaining not classified). Plots indicate data (diamonds), median (center), mean (open square), Q1/Q3 (box), and ±1.5 SD (whiskers). (**G**) Time evolution of forces exerted by three immature (top) and three mature (bottom) invadopodia. Force measurements were taken every 2.5 min; recording started 3 hours after cell seeding. Scale bars, 20 μm (A) and 1 μm (C).

Invadopodia are dynamic structures, typically showing turnover within 5 to 15 min (*40*, *41*). ERISM force measurements allowed us to study the temporal dynamics of the mechanical activity of invadopodia and to correlate them with their degradative ability. We followed the periodic protrusions of invadopodia into the substrate, using ERISM time-lapse force imaging of UM-SCC-1 cells (Fig. 3, A to C, and movies S1 and S2). From these data, we extracted the temporal evolution of the force exerted by a single invadopodium (Fig. 3D and fig. S5; a reference trace from a region not containing invadopodia corroborates that the observed changes in force are well above the noise floor of the measurement).

To analyze this behavior in more detail, we followed and analyzed the force generated by 93 invadopodia (from five different cells and three individual experiments) over the course of several hours, with data recorded every 10 s. This revealed a two-mode oscillatory behavior; on average, invadopodia showed cycles of high mechanical activity lasting 7.2 ± 4.1 min (Fig. 3E) and, in addition, exhibited a fast oscillation in force with a characteristic period of 43.3 ± 1.7 s (Fig. 3F and fig. S6). This two-mode behavior may reflect a combination of a periodic exertion of mechanical force supporting matrix degradation (slow force variation), accompanied by a faster oscillation. Similar dynamics, i.e., a combination of fast and slow oscillations, have also been observed when mapping stiffness changes of podosome protrusions using AFM (*22*). Comparing the force dynamics of invadopodia with their degrading ability showed that mature invadopodia were more active and persisted for a longer time span than immature invadopodia (Fig. 3G and movies S1 and S2). Combined with our earlier observation of larger forces in mature invadopodia, this observation suggests a link between successful ECM degradation and the presence of persistent and oscillatory forces.

### miR-375 overexpression affects invadopodia mechanics

miRNAs are small, noncoding strands of RNA, which regulate gene expression in health and malignancy and behave as oncogenes or cancer suppressors. Previous work has shown that low-level expression of miR-375 enhances cancer invasiveness and is associated with poor patient outcome (*33*); conversely, overexpression of miR-375 can suppress invadopodia maturation and reduce cancer invasiveness (*34*). Here, we investigated the effect of miR-375 overexpression on the mechanical activity of UM-SCC-1 cells.

**Fig. 4:**
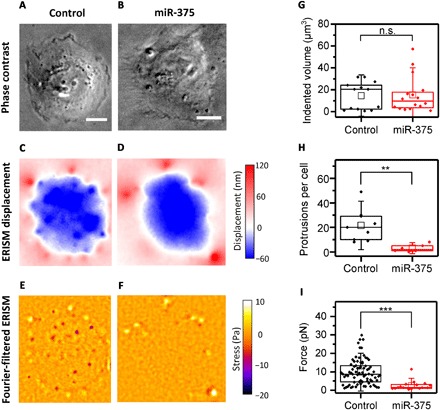
Impact of overexpression of the miR-375 on the mechanical activity of UM-SCC-1 cells. (**A** and **B**) Phase-contrast, (**C** and **D**) ERISM, and (**E** and **F**) Fourier-filtered ERISM images of representative UM-SCC-1 control cell (left column) and miR-375–overexpressing cell (right column). (**G**) Comparison of total volume by which cells indent into the ERISM substrate. Control, *n* = 13 cells; miR-375, *n* = 16 cells. As data in groups were not normally distributed, groups were compared using the Mann-Whitney *U* test (*P* = 0.983). (**H**) Comparison of the number of protruding invadopodia formed per cell (mixed population of mature and immature invadopodia). Control, *n* = 9 cells; miR-375, *n* = 6 cells. Two-sample *t* test (*P* = 0.005). (**I**) Comparison of force exerted by invadopodia from both cell types. Control, *n* = 106 invadopodia from eight cells; miR-375, *n* = 47 invadopodia from three cells. As data in groups were not normally distributed, groups were compared using the Mann-Whitney *U* test (*P* = 1.41 × 10^−6^). Statistical tests: not significant (n.s.), *P* > 0.05; **P < 0.01; ***P < 0.001. Scale bars, 20 μm.

Control and miR-375–overexpressing UM-SCC-1 cells showed comparable morphology (Fig. 4, A and B). Likewise, the broad indentation across the cell body and the upward pulling along the cell periphery were comparable (Fig. 4, C and D). However, after filtering out the broad indentation using spatial Fourier filtering as described before, we found that miR-375 overexpression led to a significant change in the mechanical activity of individual invadopodia with fewer and weaker invadopodia (Fig. 4, E and F). To quantify the effect of miR-375 overexpression on mechanical activity, we first calculated the total volume by which cells indent into the substrate, which provides a measure of the overall contractile force generated by the whole cell (Fig. 4G). This showed no significant difference between the two cell populations. However, the number of indenting invadopodia per cell in miR-375–overexpressing cells decreased significantly (Fig. 4H), and the force exerted by the remaining invadopodia became significantly smaller (Fig. 4I). Earlier findings showed that overexpression of miR-375 in UM-SCC-1 cells decreases the proteolytic behavior of these cells, i.e., fewer invadopodia and less invasion, but that the remaining invadopodia stained positively for Tks5 and that some invadopodia retained degrading ability (*34*). Our experiments now show that miR-375 overexpression also compromises invadopodia mechanics, consistent with a block in invadopodia maturation.

## DISCUSSION

On the basis of the results of our investigation, we propose a mechanical model for invadopodia-mediated cancer invasion. Formation of an invadopodium is followed by actin polymerization in the main invadopodium core, which then leads to a protrusion at the ventral part of the cell membrane (*42*). These protrusions exert piconewton pushing forces, i.e., forces that are directed perpendicular and outward with respect to the cell membrane. Mature (degrading) invadopodia apply, on average, significantly higher forces than immature (nondegrading) ones.

There is increasing interest in the temporal dynamics of cell forces, e.g., to understand whether and how mechanical aspects regulate cell cycle progression (*43*). Our work now shows that invadopodial forces are also highly dynamic with fluctuations on two distinct time scales; they exert a vertical downward force about every 7 min, which is accompanied by faster variations in force approximately every 40 s. We hypothesize that the slow component of the dynamics is associated with actin polymerization at the invadopodium core, which, after a few minutes, reaches its maximum length, causing the invadopodium to collapse and actin monomers to be released back into the cytoplasm, thereby triggering a new cycle of actin polymerization and, thus, invadopodium formation (*40*, *41*). The faster force fluctuations may instead be due to treadmilling of actin filaments at the invadopodium core (*44*) and could be associated with mechanosensing and membrane tension (*45*–*48*).

Invadopodial forces are more dynamic and persistent in mature invadopodia, suggesting that the protrusive activity (i.e., the slow force component) contributes to matrix degradation and to breaking of the ECM barrier. Protrusion force could expose sites in the ECM that could be remodeled or bring membrane-associated proteases such as MT1-MMP into close contact with ECM substrates, resulting in efficient matrix degradation and cancer invasion. The difference in force exerted by mature and immature invadopodia further suggests that there may be a minimum threshold force for invadopodia to achieve ECM degradation.

In agreement with earlier studies, we observed that overexpression of miR-375 significantly decreases the number of invadopodia (*33*, *34*). However, in addition, we found that the forces generated by the few surviving invadopodia were markedly decreased but that mechanical activity and contractility on the level of the whole cell were not affected. This indicates that miR-375 overexpression specifically impairs the formation and function of invadopodia, thus providing a potential avenue for reducing invadopodia-mediated invasion and metastasis in cancer therapy.

In the future, studying the effect of external mechanical and chemical signals on invadopodial force is likely to improve our understanding of invadopodia-mediated cancer invasion. On the basis of our observations and measurements, future studies should attempt to further elucidate the connection between mechanical forces exerted by invadopodia and their ability for ECM degradation, particularly to establish if there is a causal link and a defined timing of events, i.e., if force precedes or follows ECM degradation. It will also be interesting to study the mechanical activity and proteolytic ability upon silencing, overexpression, or mutation of invadopodia-associated proteins, such as cortactin or MMPs. In addition, ERISM may provide new insights into the role of invadopodia in cell adhesion, which is believed to play a crucial role in cancer. The invadopodium core is surrounded by adhesion proteins (e.g., paxillin and vinculin), which create a ring-like structure around the main actin core (*49*), and it is tempting to speculate that this adhesive structure exerts forces that counterbalance the protrusion of the invadopodium core or influence the mechanical activity of invadopodia in other ways.

## MATERIALS AND METHODS

### Cell lines and cell cultures

Cells of the HNSCC cell line UM-SCC-1 and a previously developed derivative, in which the miR-375 is overexpressed (*33*), were provided by T. Carey from the University of Michigan. Cells were cultured in Dulbecco’s modified Eagle’s medium (DMEM; high glucose, GlutaMAX-I, Gibco 61965-026) fortified with 10 volume % fetal bovine serum (Biosera FB-1001), 1 volume % minimum essential medium, nonessential amino acids (100×, Gibco 11140-035), and 1 volume % penicillin-streptomycin (Gibco 15140122). For epifluorescence microscopy, phenol red–free DMEM (Gibco 31053-028) with 1 volume % GlutaMAX-I was used.

ERISM substrates were fabricated as described previously (*23*). The substrate surface was separated into smaller compartments by mounting 7.5 × 7.5–mm^2^ silicone chambers on the ERISM substrate (obtained by cutting down removable 12-well chambers; Ibidi 81201), and 3000 cells were plated in each chamber.

### Collagen-I coating

Collagen-I (1 mg/ml; Millipore L7220) was mixed with low-pH phosphate-buffered saline (PBS) (pH 3.0 to 3.5) in a 1:1 ratio, at a total volume of 350 μl for each silicone chamber. The mixture was applied to the ERISM substrate and incubated for 30 min at 37°C and 5% CO_2_. The substrate was then washed three times with PBS (pH 7.4) and twice with cell culture medium.

### Gelatin coating

Gelatin coatings on ERISM substrates were prepared using a commercial assay (Millipore ECM670) following the manufacturer’s instructions, except that during the washing steps, the ERISM substrate was not allowed to fall completely dry to prevent damage to the surface. To still ensure complete removal of the previous reagent, the number of washing steps was increased from 3 to 6. Moreover, the chamber was washed twice with each reagent before incubation to minimize dilution of the reagent by the previously present liquid. The thickness of the gelatin coating on gold was determined with an AFM (Nanosurf, FlexAFM).

### Immunostaining

Cells were fixed directly on the ERISM substrate with 4% formaldehyde (formaldehyde 37%, Millipore 1.04003.1000) in PBS for 20 min, washed twice with 0.05% Tween 20 (diluted from Alfa Aesar J63596; all washing steps involved a 5-min incubation at room temperature, unless noted otherwise), and permeabilized with 0.05% Triton X-100 (Alfa Aesar A16046). After again washing twice with 0.05% Tween 20, cells were blocked with 1% bovine serum albumin (BSA; Albumin Fraction V, >98%; Roth 80761) in PBS for 30 min, incubated with a primary anticortactin antibody (1 mg/ml; Millipore 05-180; 1:100 dilution in 1% BSA) or anti-Tks5 antibody [fish (M-300), 200 μg/ml; Santa Cruz Biotechnology sc-30122; 1:50 dilution in 1% BSA) at room temperature for 1 hour, washed three times in 0.05% Tween 20 and twice with 1% BSA, and then incubated with a secondary Cy3 anti-mouse antibody (1.5 mg/ml; Jackson ImmunoResearch Laboratories 715-165-150; 1:400 dilution in 1% BSA) and TRITC (tetramethyl rhodamine isothiocyanate)–phalloidin (EMD Millipore CS207796; 1:50 dilution in 1% BSA) or Cy5 anti-rabbit (1.5 mg/ml; Jackson ImmunoResearch Laboratories 711-175-152; 1:400 dilution in 1% BSA) at room temperature for 1 hour. Cells were again washed three times with 0.05% Tween 20 and twice with 1% BSA. Last, cells were incubated with DAPI (4′,6-diamidino-2-phenylindole) (EMD Millipore 90229; 1:100 dilution in 1% BSA) at room temperature for 3 min and washed three times with 0.05% Tween 20.

### Image analysis, data analysis, and statistical testing

ERISM displacement maps were generated from recorded sets of monochromatic interference images using an in-house Python code, and ERISM stress map was generated from these using finite element modeling (COMSOL Multiphysics) as described previously (*23*). Further image analysis was performed in ImageJ. A fast Fourier transform (FFT) filter was applied to remove unspecific broad deformations caused by focal adhesion–mediated overall contractility of the cell. FFT parameters were typically set to filter features larger than 10 pixels (1.62 μm) and smaller than 3 pixels (0.49 μm) from each stress map. To measure the invadopodia force or the total indented volume for an entire cell, a threshold was applied to the image, before integrating over areas of downward stress (for force) or downward deformation (for volume).

To quantify the period of fast oscillations, stress maps were acquired every 10 s, and the force exerted by the respective invadopodium was plotted over time. The time at which the local maximum in force occurred was read off, and the cumulative number of force maxima was plotted over time. The slope of a linear fit to these data then yielded the period at which fast force oscillations occurred. The mean of this value was calculated from analyzing multiple protrusions. For the slow force oscillation, the raw force data were again plotted over time for each protrusion, and the time difference between two force minima was read off. The mean of these values was calculated from analyzing multiple protrusions. Force measurements lasted 2.0 to 4.5 hours.

Statistical testing was performed with the OriginPro 2017 software (OriginLab Corp). For normally distributed data, the two-sample *t* test was applied, whereas the Mann-Whitney nonparametric test was used when the data were not normally distributed. The level of significance was set to 0.05, and the calculated *P* value was used to define the significant difference between the compared populations.

## Supplementary Material

aax6912_Movie_S2.avi

aax6912_Movie_S1.avi

aax6912_SM.pdf

## SUPPLEMENTARY MATERIALS

Supplementary material for this article is available at http://advances.sciencemag.org/cgi/content/full/6/11/eaax6912/DC1 Fig. S1. AFM measurement of thickness and apparent stiffness of ERISM substrate and gelatin coating. Fig. S2. ERISM and immunostaining for actin, cortactin, and Tks5 in UM-SCC-1 cells. Fig. S3. Simultaneous imaging of gelatin matrix degradation and invadopodia force. Fig. S4. Time dependence of invadopodia force within a single cell. Fig. S5. Long-term measurement of invadopodia forces. Fig. S6. Fourier transform–based analysis of the characteristic period of force oscillation in invadopodia. Movie S1. Temporal evolution of invadopodia force for a typical cell with immature invadopodia. Movie S2. Temporal evolution of invadopodia force for a typical cell with predominantly mature invadopodia.

## Appendix

View/request a protocol for this paper from *Bio-protocol*.

## References

[1] Northcott J. M., Dean I. S., Mouw J. K., Weaver V. M., Feeling stress: The mechanics of cancer progression and aggression. Front. Cell Dev. Biol. 6, 17 (2018). 29541636 10.3389/fcell.2018.00017 PMC5835517

[2] Parekh A., Weaver A. M., Regulation of cancer invasiveness by the physical extracellular matrix environment. Cell Adh. Migr. 3, 288–292 (2009). 19458499 10.4161/cam.3.3.8888 PMC2712813

[3] Remmerbach T. W., Wottawah F., Dietrich J., Lincoln B., Wittekind C., Guck J., Oral cancer diagnosis by mechanical phenotyping. Cancer Res. 69, 1728–1732 (2009). 19223529 10.1158/0008-5472.CAN-08-4073

[4] Linder S., Wiesner C., Himmel M., Degrading devices: Invadosomes in proteolytic cell invasion. Annu. Rev. Cell Dev. Biol. 27, 185–211 (2011). 21801014 10.1146/annurev-cellbio-092910-154216

[5] Eddy R. J., Weidmann M. D., Sharma V. P., Condeelis J. S., Tumor cell invadopodia: Invasive protrusions that orchestrate metastasis. Trends Cell Biol. 27, 595–607 (2017). 28412099 10.1016/j.tcb.2017.03.003 PMC5524604

[6] Murphy D. A., Courtneidge S. A., The ‘ins’ and ‘outs’ of podosomes and invadopodia: Characteristics, formation and function. Nat. Rev. Mol. Cell Biol. 12, 413–426 (2011). 21697900 10.1038/nrm3141 PMC3423958

[7] Albiges-Rizo C., Destaing O., Fourcade B., Planus E., Block M. R., Actin machinery and mechanosensitivity in invadopodia, podosomes and focal adhesions. J. Cell Sci. 122, 3037–3049 (2009). 19692590 10.1242/jcs.052704 PMC2767377

[8] Beaty B. T., Condeelis J., Digging a little deeper: The stages of invadopodium formation and maturation. Eur. J. Cell Biol. 93, 438–444 (2014). 25113547 10.1016/j.ejcb.2014.07.003 PMC4262566

[9] Artym V. V., Zhang Y., Seillier-Moiseiwitsch F., Yamada K. M., Mueller S. C., Dynamic interactions of cortactin and membrane type 1 matrix metalloproteinase at invadopodia: Defining the stages of invadopodia formation and function. Cancer Res. 66, 3034–3043 (2006). 16540652 10.1158/0008-5472.CAN-05-2177

[10] Kirkbride K. C., Sung B. H., Sinha S., Weaver A. M., Cortactin: A multifunctional regulator of cellular invasiveness. Cell Adh. Migr. 5, 187–198 (2011). 21258212 10.4161/cam.5.2.14773 PMC3084985

[11] Ren X. L., Qiao Y. D., Li J. Y., Li X. M., Zhang D., Zhang X. J., Zhu X. H., Zhou W. J., Shi J., Wang W., Liao W. T., Ding Y. Q., Liang L., Cortactin recruits FMNL2 to promote actin polymerization and endosome motility in invadopodia formation. Cancer Lett. 419, 245–256 (2018). 29374558 10.1016/j.canlet.2018.01.023

[12] Iizuka S., Abdullah C., Buschman M. D., Diaz B., Courtneidge S. A., The role of Tks adaptor proteins in invadopodia formation, growth and metastasis of melanoma. Oncotarget 7, 78473–78486 (2016). 27802184 10.18632/oncotarget.12954 PMC5346654

[13] Linder S., Scita G., RABGTPases in MT1-MMP trafficking and cell invasion: Physiology versus pathology. Small GTPases 6, 145–152 (2015). 26107110 10.4161/21541248.2014.985484 PMC4601268

[14] Gasparski A. N., Ozarkar S., Beningo K. A., Transient mechanical strain promotes the maturation of invadopodia and enhances cancer cell invasion in vitro. J. Cell Sci. 130, 1965–1978 (2017). 28446539 10.1242/jcs.199760 PMC6865334

[15] Magalhaes M. A. O., Larson D. R., Mader C. C., Bravo-Cordero J. J., Gil-Henn H., Oser M., Chen X., Koleske A. J., Condeelis J., Cortactin phosphorylation regulates cell invasion through a pH-dependent pathway. J. Cell Biol. 195, 903–920 (2011). 22105349 10.1083/jcb.201103045 PMC3257566

[16] Ouderkirk J. L., Krendel M., Myosin 1e is a component of the invadosome core that contributes to regulation of invadosome dynamics. Exp. Cell Res. 322, 265–276 (2014). 24462457 10.1016/j.yexcr.2014.01.015 PMC4019408

[17] Alexander N. R., Branch K. M., Parekh A., Clark E. S., Iwueke I. C., Guelcher S. A., Weaver A. M., Extracellular matrix rigidity promotes invadopodia activity. Curr. Biol. 18, 1295–1299 (2008). 18718759 10.1016/j.cub.2008.07.090 PMC2555969

[18] Jerrell R. J., Parekh A., Cellular traction stresses mediate extracellular matrix degradation by invadopodia. Acta Biomater. 10, 1886–1896 (2014). 24412623 10.1016/j.actbio.2013.12.058 PMC3976707

[19] Aung A., Seo Y. N., Lu S., Wang Y., Jamora C., del Álamo J. C., Varghese S., 3D traction stresses activate protease-dependent invasion of cancer cells. Biophys. J. 107, 2528–2537 (2014). 25468332 10.1016/j.bpj.2014.07.078 PMC4255420

[20] Revach O.-Y., Weiner A., Rechav K., Sabanay I., Livne A., Geiger B., Mechanical interplay between invadopodia and the nucleus in cultured cancer cells. Sci. Rep. 5, 9466 (2015). 25820462 10.1038/srep09466 PMC4377574

[21] Labernadie A., Bouissou A., Delobelle P., Balor S., Voituriez R., Proag A., Fourquaux I., Thibault C., Vieu C., Poincloux R., Charrière G. M., Maridonneau-Parini I., Protrusion force microscopy reveals oscillatory force generation and mechanosensing activity of human macrophage podosomes. Nat. Commun. 5, 5343 (2014). 25385672 10.1038/ncomms6343

[22] Labernadie A., Thibault C., Vieu C., Maridonneau-Parini I., Charrière G. M., Dynamics of podosome stiffness revealed by atomic force microscopy. Proc. Natl. Acad. Sci. U.S.A. 107, 21016–21021 (2010). 21081699 10.1073/pnas.1007835107 PMC3000246

[23] Kronenberg N. M., Liehm P., Steude A., Knipper J. A., Borger J. G., Scarcelli G., Franze K., Powis S. J., Gather M. C., Long-term imaging of cellular forces with high precision by elastic resonator interference stress microscopy. Nat. Cell Biol. 19, 864–872 (2017). 28628084 10.1038/ncb3561

[24] Cervero P., Wiesner C., Bouissou A., Poincloux R., Linder S., Lymphocyte-specific protein 1 regulates mechanosensory oscillation of podosomes and actin isoform-based actomyosin symmetry breaking. Nat. Commun. 9, 515 (2018). 29410425 10.1038/s41467-018-02904-x PMC5802837

[25] Rafiq N. B. M., Lieu Z. Z., Jiang T., Yu C.-H., Matsudaira P., Jones G. E., Bershadsky A. D., Podosome assembly is controlled by the GTPase ARF1 and its nucleotide exchange factor ARNO. J. Cell Biol. 216, 181–197 (2016). 28007915 10.1083/jcb.201605104 PMC5223603

[26] Kedziora K. M., Isogai T., Jalink K., Innocenti M., Invadosomes—Shaping actin networks to follow mechanical cues. Front. Biosci. (Landmark Ed.) 21, 1092–1117 (2016). 27100494 10.2741/4444

[27] Wolf K., Friedl P., Extracellular matrix determinants of proteolytic and non-proteolytic cell migration. Trends Cell Biol. 21, 736–744 (2011). 22036198 10.1016/j.tcb.2011.09.006

[28] Maskarinec S. A., Franck C., Tirrell D. A., Ravichandran G., Quantifying cellular traction forces in three dimensions. Proc. Natl. Acad. Sci. U.S.A. 106, 22108–22113 (2009). 20018765 10.1073/pnas.0904565106 PMC2799761

[29] Plotnikov S. V., Sabass B., Schwarz U. S., Waterman C. M., High-resolution traction force microscopy. Methods Cell Biol. 123, 367–394 (2014). 24974038 10.1016/B978-0-12-420138-5.00020-3 PMC4699589

[30] Ghassemi S., Meacci G., Liu S., Gondarenko A. A., Mathur A., Roca-Cusachs P., Sheetz M. P., Hone J., Cells test substrate rigidity by local contractions on submicrometer pillars. Proc. Natl. Acad. Sci. U.S.A. 109, 5328–5333 (2012). 22431603 10.1073/pnas.1119886109 PMC3325713

[31] Polacheck W. J., Chen C. S., Measuring cell-generated forces: A guide to the available tools. Nat. Methods 13, 415–423 (2016). 27123817 10.1038/nmeth.3834 PMC5474291

[32] Liehm P., Kronenberg N. M., Gather M. C., Analysis of the precision, robustness, and speed of elastic resonator interference stress microscopy. Biophys. J. 114, 2180–2193 (2018). 29742411 10.1016/j.bpj.2018.03.034 PMC5961519

[33] Harris T., Jimenez L., Kawachi N., Fan J.-B., Chen J., Belbin T., Ramnauth A., Loudig O., Keller C. E., Smith R., Prystowsky M. B., Schlecht N. F., Segall J. E., Childs G., Low-level expression of miR-375 correlates with poor outcome and metastasis while altering the invasive properties of head and neck squamous cell carcinomas. Am. J. Pathol. 180, 917–928 (2012). 22234174 10.1016/j.ajpath.2011.12.004 PMC3349885

[34] Jimenez L., Sharma V. P., Condeelis J., Harris T., Ow T. J., Prystowsky M. B., Childs G., Segall J. E., MicroRNA-375 suppresses extracellular matrix degradation and invadopodial activity in head and neck squamous cell carcinoma. Arch. Pathol. Lab. Med. 139, 1349–1361 (2015). 26172508 10.5858/arpa.2014-0471-OA PMC4628565

[35] Leemans C. R., Snijders P. J. F., Brakenhoff R. H., The molecular landscape of head and neck cancer. Nat. Rev. Cancer 18, 269–282 (2018). 29497144 10.1038/nrc.2018.11

[36] Siegel R. L., Miller K. D., Jemal A., Cancer statistics, 2018. CA Cancer J. Clin. 68, 7–30 (2018). 29313949 10.3322/caac.21442

[37] Cheng S., Gandevia S. C., Green M., Sinkus R., Bilston L. E., Viscoelastic properties of the tongue and soft palate using MR elastography. J. Biomech. 44, 450–454 (2011). 21040923 10.1016/j.jbiomech.2010.09.027

[38] Herman J., Sedlackova Z., Vachutka J., Furst T., Salzman R., Vomacka J., Shear wave elastography parameters of normal soft tissues of the neck. Biomed. Pap. Med. Fac. Univ. Palacky Olomouc Czech. Repub. 161, 320–325 (2017). 28546639 10.5507/bp.2017.024

[39] Pourfarhangi K. E., Bergman A., Gligorijevic B., ECM cross-linking regulates invadopodia dynamics. Biophys. J. 114, 1455–1466 (2018). 29590602 10.1016/j.bpj.2018.01.027 PMC5883616

[40] Sharma V. P., Entenberg D., Condeelis J., High-resolution live-cell imaging and time-lapse microscopy of invadopodium dynamics and tracking analysis. Methods Mol. Biol. 1046, 343–357 (2013). 23868599 10.1007/978-1-62703-538-5_21 PMC3933219

[41] Jimenez L., Jayakar S. K., Ow T. J., Segall J. E., Mechanisms of invasion in head and neck cancer. Arch. Pathol. Lab. Med. 139, 1334–1348 (2015). 26046491 10.5858/arpa.2014-0498-RA PMC7469951

[42] Uroz M., Wistorf S., Serra-Picamal X., Conte V., Sales-Pardo M., Roca-Cusachs P., Guimerà R., Trepat X., Regulation of cell cycle progression by cell–cell and cell–matrix forces. Nat. Cell Biol. 20, 646–654 (2018). 29802405 10.1038/s41556-018-0107-2

[43] Baldassarre M., Ayala I., Beznoussenko G., Giacchetti G., Machesky L. M., Luini A., Buccione R., Actin dynamics at sites of extracellular matrix degradation. Eur. J. Cell Biol. 85, 1217–1231 (2006). 17010475 10.1016/j.ejcb.2006.08.003

[44] Carlier M. F., Shekhar S., Global treadmilling coordinates actin turnover and controls the size of actin networks. Nat. Rev. Mol. Cell Biol. 18, 389–401 (2017). 28248322 10.1038/nrm.2016.172

[45] Pontes B., Monzo P., Gole L., Le Roux A.-L., Kosmalska A. J., Tam Z. Y., Luo W., Kan S., Viasnoff V., Roca-Cusachs P., Tucker-Kellogg L., Gauthier N. C., Membrane tension controls adhesion positioning at the leading edge of cells. J. Cell Biol. 216, 2959–2977 (2017). 28687667 10.1083/jcb.201611117 PMC5584154

[46] Wang C., Choi H. J., Kim S.-J., Desai A., Lee N., Kim D., Bae Y., Lee K., Deconvolution of subcellular protrusion heterogeneity and the underlying actin regulator dynamics from live cell imaging. Nat. Commun. 9, 1688 (2018). 29703977 10.1038/s41467-018-04030-0 PMC5923236

[47] Huang C.-H., Tang M., Shi C., Iglesias P. A., Devreotes P. N., An excitable signal integrator couples to an idling cytoskeletal oscillator to drive cell migration. Nat. Cell Biol. 15, 1307–1316 (2013). 24142103 10.1038/ncb2859 PMC3838899

[48] Linder S., Wiesner C., Feel the force: Podosomes in mechanosensing. Exp. Cell Res. 343, 67–72 (2016). 26658516 10.1016/j.yexcr.2015.11.026

[49] Branch K. M., Hoshino D., Weaver A. M., Adhesion rings surround invadopodia and promote maturation. Biol. Open 1, 711–722 (2012). 23213464 10.1242/bio.20121867 PMC3507228

