# Supplementary material for: Direct measurement of vertical forces shows correlation between mechanical activity and proteolytic ability of invadopodia
Source: Sci Adv. 2020 Mar 11;6(11):eaax6912. doi: 10.1126/sciadv.aax6912 (PMC7065877; doi:10.1126/sciadv.aax6912)
Supplement: aax6912_SM.pdf [file aax6912_SM.pdf]

Science Advances
AAAS

advances.sciencemag.org/cgi/content/full/6/11/eaax6912/DC1

Supplementary Materials for

Direct measurement of vertical forces shows correlation between mechanical activity and proteolytic ability of invadopodia

E. Dalaka, N. M. Kronenberg, P. Liehm, J. E. Segall, M. B. Prystowsky, M. C. Gather*,

*Corresponding author. Email: mcg6@st-andrews.ac.uk

Published 11 March 2020, *Sci. Adv.* **6**, eaax6912 (2020)
DOI: 10.1126/sciadv.aax6912

The PDF file includes:

Fig. S1. AFM measurement of thickness and apparent stiffness of ERISM substrate and gelatin coating.
Fig. S2. ERISM and immunostaining for actin, cortactin, and Tks5 in UM-SCC-1 cells.
Fig. S3. Simultaneous imaging of gelatin matrix degradation and invadopodia force.
Fig. S4. Time dependence of invadopodia force within a single cell.
Fig. S5. Long-term measurement of invadopodia forces.
Fig. S6. Fourier transform–based analysis of the characteristic period of force oscillation in invadopodia.
Legends for movies S1 and S2

Other Supplementary Material for this manuscript includes the following:

(available at advances.sciencemag.org/cgi/content/full/6/11/eaax6912/DC1)

Movie S1 (.avi format). Temporal evolution of invadopodia force for a typical cell with immature invadopodia.
Movie S2 (.avi format). Temporal evolution of invadopodia force for a typical cell with predominantly mature invadopodia.

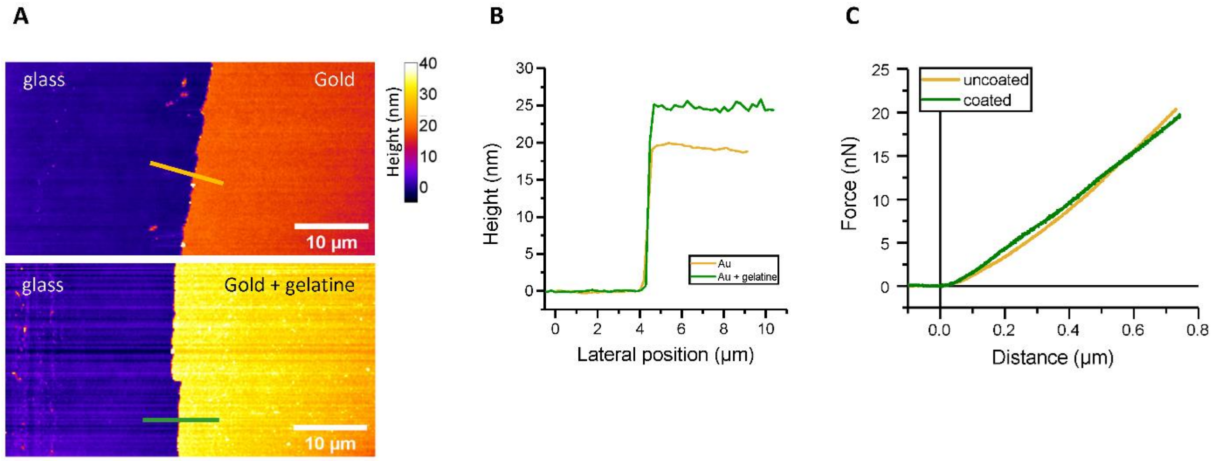

Fig. S1. AFM measurement of thickness and apparent stiffness of ERISM substrate and gelatin coating. (**A**) AFM topography maps of a scratch made along a glass substrate coated with 20 nm of gold and partially coated with fluorescent gelatine. Scratching removed both the gold and the gelatine and revealed the glass substrate underneath. The difference in step height between the area coated only with gold (19.3 ± 0.6 nm, top panel) and the area coated with gold and gelatine (24.8 ± 1.1 nm, bottom panel) corresponds to the thickness of the gelatine film (5.5 ± 1.2 nm). (**B**) Thickness profiles along the yellow and green lines of the topography maps in A. (**C**) Typical AFM force-distance curves for an uncoated ERISM substrate (yellow line) and a gelatine coated ERISM substrate (green line). The good overlap of both traces indicates that the gelatine coating does not affect the apparent stiffness of the ERISM substrate.

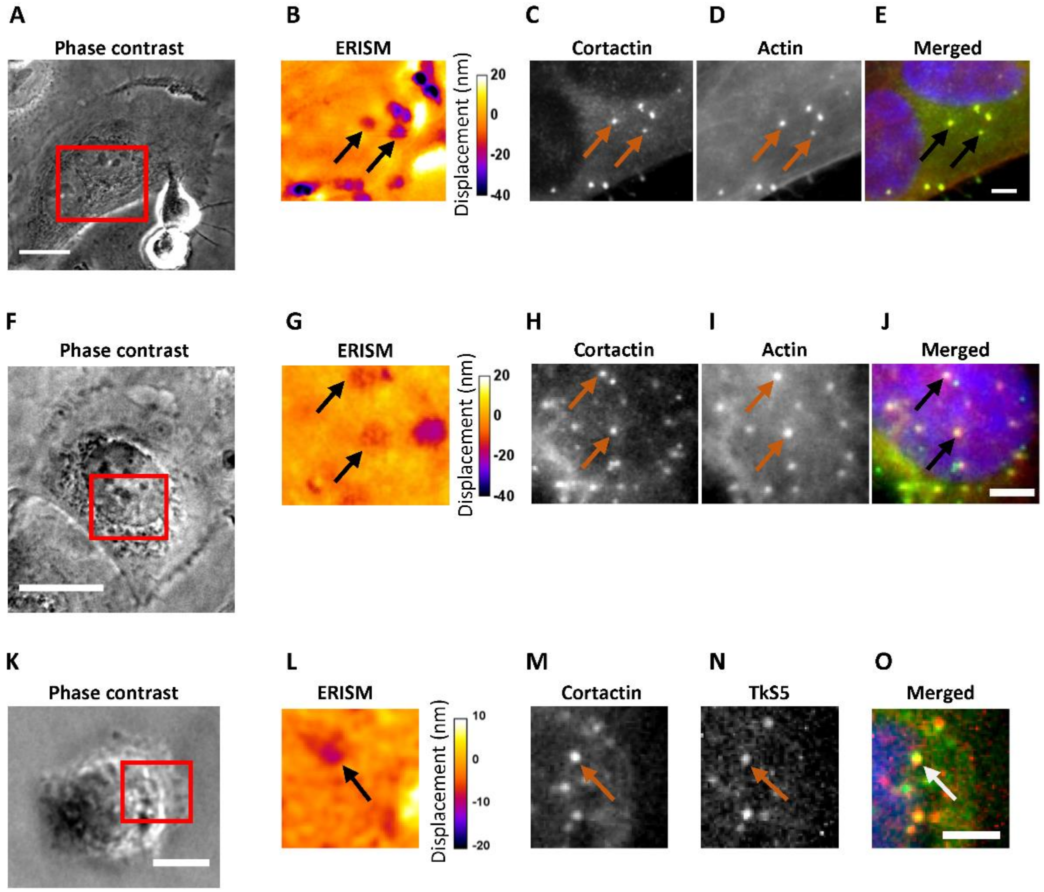

Fig. S2. ERISM and immunostaining for actin, cortactin, and Tks5 in UM-SCC-1 cells. (**A, F and K**) Phase contrast image of three representative cells, (**B, G and L**) ERISM displacement maps, (**C, H and M**) cortactin staining, (**D** and **I**) actin staining, (**N**) TkS5 staining, and (**E, J and O**) merged image of cortactin (green), actin or TkS5 (red) and DAPI (blue). The arrows indicate the correlation between actin and cortactin/TkS5 puncta and local indentations on the ERISM maps. Scale bars, 20 µm (A,F), 5 µm (B-E, G-J, L-O), 10 µm (K).

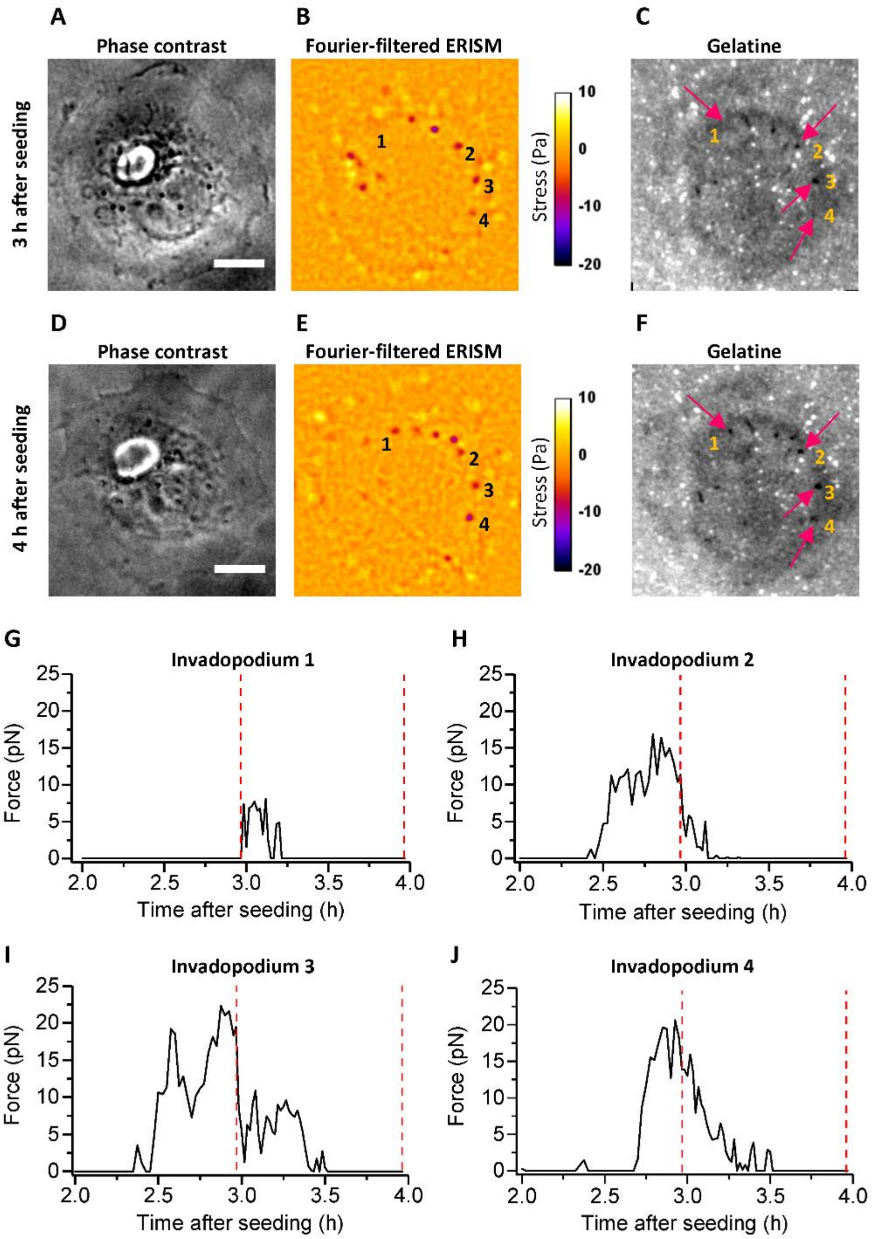

Fig. S3. Simultaneous imaging of gelatin matrix degradation and invadopodia force. (**A** and **D**) Phase contrast images, (**B** and **E**) Fourier-filtered ERISM stress maps and (**C** and **F**) epi-fluorescence image of the gelatine film on the surface of the ERISM substrate for same cell at 3 h (top images) and 4 h (bottom images) after cell seeding. The arrows in C and F indicate the matrix degradation induced by the four numbered invadopodia. ERISM stress maps were recorded continuously at 1 frame/min from 2 h after cell seeding but recording of gelatine fluorescence was limited to two images to minimize photobleaching and photodamage. Invadopodia 2 and 3 exerted force at the 3 h and 4 h time points and gelatine degradation was already visible at 3 h. The degradation spot associated with invadopodium 2 was larger after 4 h than after 3 h. Invadopodium 1 was not visible in the ERISM map at 3h and there was no associated gelatine degradation at 3 h, but after the 3 h time-point a force was registered for this invadopodium and there was a new gelatine degradation site at this location at 4 h. Invadopodium 4 exerted some force before the 3 h time point but with no or minimal degradation while there was clear force exertion and degradation at this site after 4 h. (**G** to **J**) Plots of force exerted over time for the 4 invadopodia indicated on ERISM stress maps and gelatine images above. The red dashed lines indicate the 3 h and 4 h time points of the images in A to F. Scale bars, 10 µm.

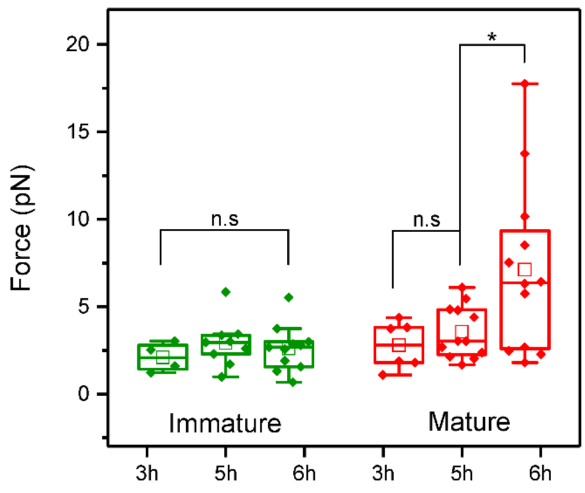

Fig. S4. Time dependence of invadopodia force within a single cell. Forces exerted by immature (green) and mature (red) invadopodia were compared at different time points (3, 5 and 6 hours) after cell seeding. Mature invadopodia become significantly stronger as time progresses while immature invadopodia show no significant change in the exerted force over time.

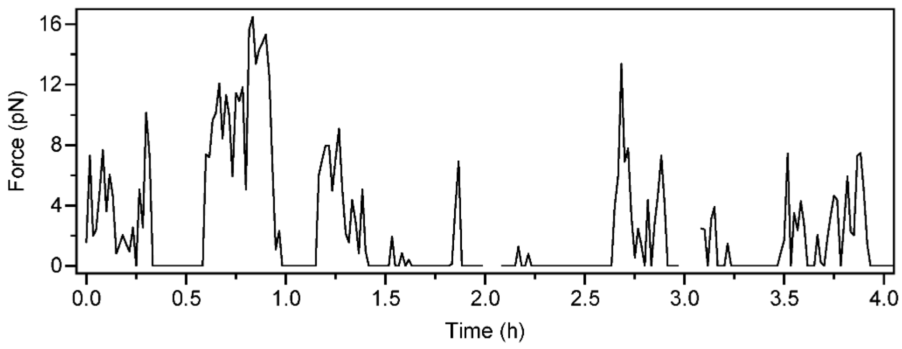

Fig. S5. Long-term measurement of invadopodia forces. Plot of force over time for a mature invadopodium. The gaps in the trace result from short interruptions of the measurements, during which no data was recorded. Intermediate periods with zero force indicate temporary disassembly and subsequent reassembly of the invadopodium. The force measurement was started 1.5 h after cell seeding.

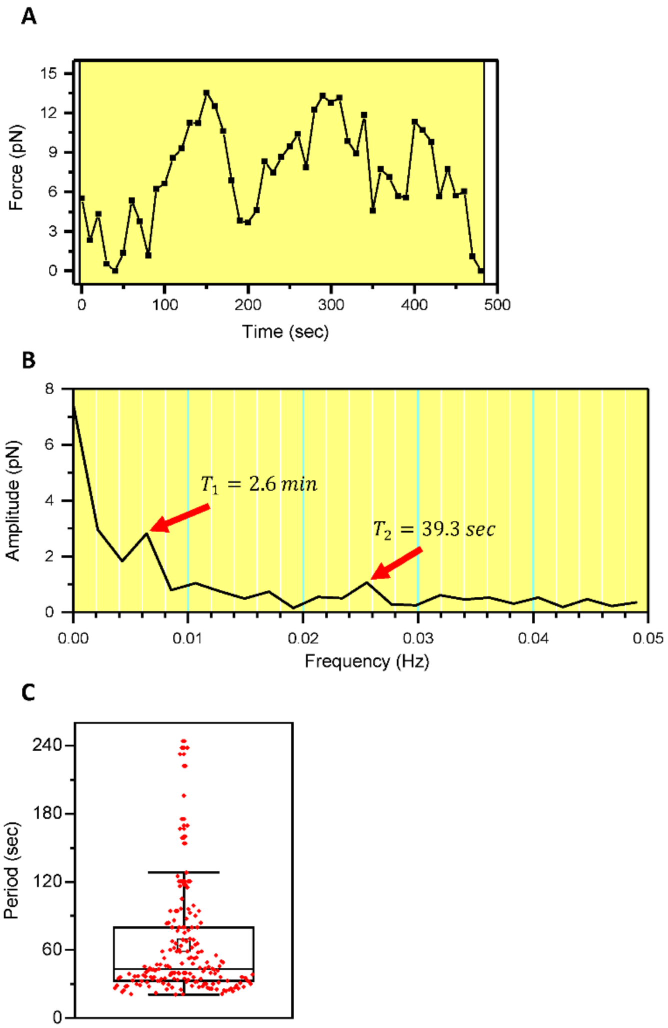

Fig. S6. Fourier transform–based analysis of the characteristic period of force oscillation in invadopodia. (**A**) Typical evolution of invadopodia force over an 8 min time course. Data recorded at 10 s per frame. Dynamic force oscillations are observed, with both slow and fast events. (**B**) FT of the data in A decomposes the signal into its different frequencies. The red arrows indicate peaks in the FT, which correspond to the predominant oscillation frequencies for the data set in A. (**C**) Boxplot analysis of the predominant oscillation frequencies found from analysing 8 min time lapse data for 62 separate invadopodia. Data from 7 cells and 2 independent experiments. The median for the invadopodia oscillation is 43.0 s, in good agreement with the method of analysing oscillation frequency used in the main text (Fig. 3F and Materials & Methods).

Movie S1. Temporal evolution of invadopodia force for a typical cell with immature invadopodia. Time-lapse of phase contrast microscopy (left) and Fourier-filtered ERISM stress maps (middle). Integrated force of the two invadopodia marked by circles in the ERISM map versus time (right). Scale bar, 10 µm.

Movie S2. Temporal evolution of invadopodia force for a typical cell with predominantly mature invadopodia. Time-lapse of phase contrast microscopy (left) and Fourier-filtered ERISM stress maps (middle). Integrated force of the two invadopodia marked by circles in ERISM map versus time (right). Scale bar, 10 µm.
